# Healthcare Disparities and Upper Gastrointestinal Bleeding: Exploring the Linkages

**DOI:** 10.7759/cureus.37977

**Published:** 2023-04-22

**Authors:** Rajmohan Rammohan, Melvin V Joy, Tulika Saggar, Sai Greeshma Magam, Atul Sinha, Dilman Natt, Sandra Gomez, Saher Sheikh, Prachi Anand, Paul Mustacchia

**Affiliations:** 1 Gastroenterology, Nassau University Medical Center, East Meadow, USA; 2 Internal Medicine, Nassau University Medical Center, East Meadow, USA; 3 Gastroenterology and Hepatology, Nassau University Medical Center, East Meadow, USA; 4 Rheumatology, Nassau University Medical Center, East Meadow, USA

**Keywords:** upper gastrointestinal (ugi) bleeding, healthcare facilities, socioeconomic factors, racial and ethnic disparities, health equity

## Abstract

Introduction

Upper gastrointestinal bleeding (UGIB) refers to blood loss from a gastrointestinal (GI) source proximal or above the ligament of Treitz. Health equity means giving everyone an equal chance to achieve optimal health by addressing injustices, overcoming barriers, and eliminating health disparities. Healthcare providers must analyze racial and ethnic disparities in UGIB management to ensure all patients receive equal care. Identifying risk factors in specific populations leads to tailored interventions that improve outcomes. Our study aims to examine trends and identify disparities in upper gastrointestinal bleeding across races and ethnicities to promote health equity.

Methods

Retrospective data on upper gastrointestinal bleeding from June 2009 to June 2022 were collected and categorized into five groups based on race. The baseline characteristics of each group were matched to ensure equitable comparison. A joinpoint regression model was used to compare incidence trends, identifying potential healthcare disparities for different racial/ethnic groups over time. Patients aged 18-75 who suffered from upper gastrointestinal bleeding at Nassau University Medical Center in New York from 2010 to 2021 were selected, excluding those with incomplete baseline comorbidity information.

Results

This study examined 5103 cases of upper gastrointestinal bleeding, with 41.9% female. The cohort was diverse, with 29.4% African American, 15.6% Hispanic, 45.3% White, 6.8% Asian, and 2.9% of other races. Data were split into two groups; 49.9% occurred between 2009 and 2015 and 50.1% between 2016 and 2022. Findings showed increased UGIB among Hispanics and decreased bleeding among Asians during 2016-2021 compared to 2009-2015. However, no significant difference was found for African Americans, Whites, and other races. In addition, Hispanics had a rise in the annual percentage change (APC) rate, whereas Asians had a decrease.

Conclusion

Our study examined trends in upper gastrointestinal bleeding and potential healthcare disparities across races and ethnicities. Our findings highlight an increased incidence of UGIB in Hispanics and a decreased incidence in Asians. Additionally, we identified a significant increase in the annual percentage change rate in Hispanics and a decrease in Asians over time. Our study underscores the importance of identifying and addressing disparities in UGIB management to promote health equity. Future research can build on these findings to develop tailored interventions that improve patient outcomes.

## Introduction

Upper gastrointestinal bleeding (UGIB) refers to blood loss from a gastrointestinal (GI) source proximal or above the ligament of Treitz. Hematemesis, hematochezia, and melena are common manifestations of this condition [1]. Health equity is the state where everyone has an equal opportunity to achieve optimal health. Achieving health equity involves addressing historical and contemporary injustices; overcoming social, economic, and other obstacles to healthcare; and eliminating preventable health disparities [2,3]. Reducing disparities in UGIB management is essential to ensure all patients receive equal care and access to effective treatments, irrespective of race or ethnicity. By analyzing racial and ethnic disparities in UGIB, healthcare providers can customize their diagnostic and treatment approaches to improve outcomes. Identifying risk factors more common in certain racial/ethnic groups can also lead to tailored interventions for these populations.

Disparities in upper GI bleeding can lead to higher rates of hospitalizations, more extended hospital stays, and higher healthcare costs, affecting health outcomes significantly. For instance, minorities who face difficulty accessing healthcare services may experience delayed diagnosis and treatment, resulting in complications from UGIB [4]. Studies have shown that gastrointestinal ulcer disease is the most common cause of bleeding or melena in patients younger than 35 or older than 65, regardless of ethnicity. However, differences in the etiology of melena were observed among patients aged 35-64 years. For instance, African Americans aged 50-64 frequently experienced gastroduodenal ulcers, while Hispanics aged 35-49 typically had esophageal varices. In addition, rebleeding rates were significantly lower in Whites (5.8%) than in Hispanics (9.9%) or African Americans (8.7%) [4,5].

Another study conducted in North Carolina hospitals [6] with a representative sample of the US population showed that despite an overall decrease in bleeds during the COVID-19 period, racial minorities experienced an absolute increase in the most severe bleeds, requiring intensive care unit (ICU) care and multiple transfusions. This disparity was not observed in non-Hispanic White patients during the same period.

Our study aims to examine UGIB trends across various races and ethnicities, with a secondary focus on addressing disparities. By doing so, we aim to reduce health inequalities and improve overall health outcomes.

This article has been accepted for presentation at Digestive Disease Week (DDW) conference in May 2023 in Chicago.

## Materials and methods

Study design

Data on upper gastrointestinal bleeding were retrospectively collected from June 2009 to June 2021, categorizing patients into five groups based on race. Before analyzing the results, the baseline characteristics of each group were matched to ensure equitable comparison. In addition, a joinpoint regression model was used to compare incidence trends between 2009-2015 and 2016-2021 regarding race/ethnicity. This approach enables researchers to examine the effects of race/ethnicity on incidence rates of UGIB over time. In addition, it helps to identify potential disparities in healthcare access or outcomes for different racial/ethnic groups.

Data source

Data were collected retrospectively using the Sunrise Electronic Medical Record software (Allscripts, Chicago, IL). The data collected included detailed information such as age, race, gender, 30-day mortality, length of stay, medication, alcohol abuse, liver disorders, upper GI bleeding, and comorbidities. This information was obtained using Current Procedural Terminology (CPT) 10 codes, including K92.2, K29.71, I86.4, I10, 0W3P4ZZ, 0W3P8, 0W3P8ZZ, 0W3P7ZZ, I63.9, D64.9, K70.3, K74.4, K74.5, K25.9, E08, E78.5, and K21.9.

Inclusion and exclusion criteria

All patients aged 18-75 who suffered from upper gastrointestinal bleeding at Nassau University Medical Center in East Meadow, New York, from 2009 to 2021 were selected for the study. However, patients with incomplete baseline comorbidity information were excluded from the study. This was likely done to ensure that the study results were not confounded by the effects of any unmeasured or unknown comorbidities that could have affected the patients' outcomes.

Analysis

Data were analyzed using Statistical Package for Social Sciences (SPSS) for Windows (IBM SPSS Statistics, Armonk, NY), RStudio software (RStudio, PBC, Boston, MA), and Joinpoint version 4.9.1.0, desktop version (National Cancer Institute, Bethesda, MD). Patient numbers (n) were used to present categorical data, while continuous data were presented as mean ± standard deviation.

## Results

This study involved 5103 cases of upper gastrointestinal bleeding, with 2142 (41.9%) female cases. The participants were individuals from various races, with 29.4% African American, 15.6% Hispanic, 45.3% White, 6.8% Asian, and 2.9% belonging to another race. The data were divided into two groups, with 49.9% of the UGIB cases occurring between 2009 and 2015 and the remaining 50.1% between 2016 and 2021. The average age in the study cohort was 67 ± 0.5 years, and Table 1 provides information on the cohort characteristics.

**Table 1:** Cohort characteristics Values are presented as numbers (n) and mean ± standard deviation

	N = 5103
Age in years	67 ± 0.5
Sex	
Male	2141 (41.3%)
Female	2962 (58%)
Coronary artery disease (CAD)	463 (9.1%)
Stroke	121 (2.4%)
Hypertension (HTN)	1635 (32%)
Diabetes	883 (17.3%)
Gastric esophageal reflux disorder	484 (9.5%)
Liver disease	1759 (34.4%)
Heart failure	205 (4%)
Chronic kidney disease	320 (6.2%)
Alcohol use	1655 (32.4%)

The study revealed increased UGIB among the Hispanic population from 2016 to 2021 compared to 2009 to 2015, with 275 cases (34.5%) in 2016-2021 and 520 cases (65.4%) in 2009-2015. On the other hand, the Asian population showed a lower number of UGIB during 2016-2021 compared to 2009-2015, with 195 cases (56.5%) and 150 cases (43.5%), respectively. However, there was no significant difference in the UGIB between the two periods for African Americans, Whites, and other races, as shown in Table 2.

**Table 2:** Upper gastrointestinal bleeding regarding race n = number of patients

Race	Total cases (n)	2009-2015 (n)	2016-2021 (n)	P-value
African American	1502	790 (52.5%)	712 (47.4%)	p = 0.457
Hispanic	795	275 (34.5%)	520 (65.4%)	p < 0.01
White	2313	1212 (52.3%)	1101(47.6%)	p = 0.447
Asian	345	195 (56.5%)	150 (43.4%)	p < 0.01
Other races	148	78 (52.7%)	70 (47.2%)	p = 0.411

**Figure 1:**
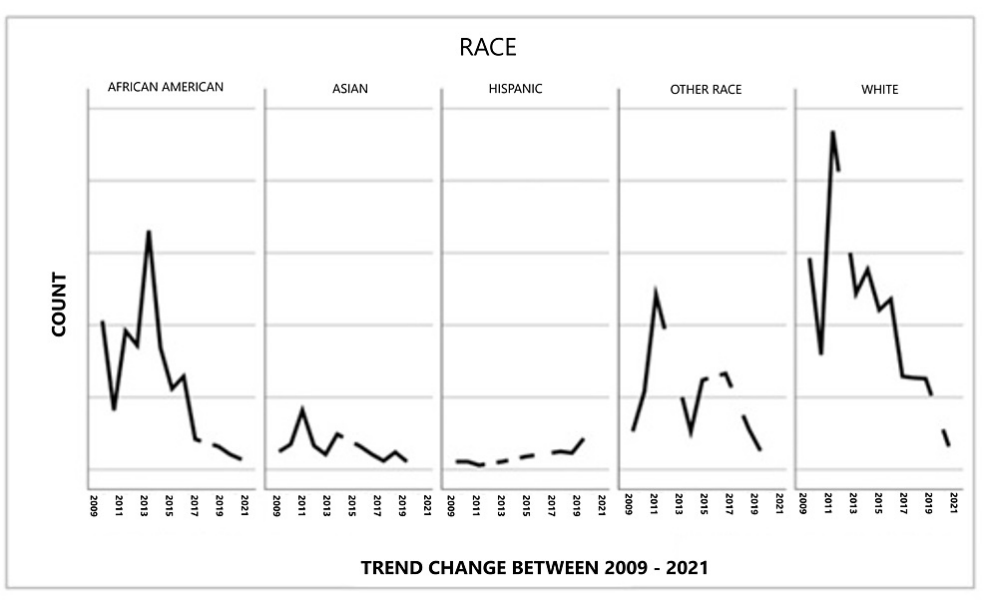
Graphical trend change regarding race between 2009 and 2021

Further analysis of the data showed that the Hispanic population exhibited an increase in the annual percentage change (APC) rate between 2009-2015 and 2016-2021 (0.3 {0.1, 0.9} versus 0.9 {0.4, 1.3}, p = 0.025). Conversely, the Asian population showed a decrease of 0.9 (0.6, 1.5) versus -1.4 (-2.1, -1.1) (p < 0.01). However, there was no significant change in the APC rate among African Americans, Whites, and other races, as shown in Table 3. A graphical representation of the trend changes in UGIB between 2009 and 2021 is shown in Figure 1.

**Table 3:** Average annual percentage change between 2009-2015 and 2016-2021 regarding race Data are represented with a 95% confidence interval; data are calculated using joinpoint regression

	2009-2015 annual percentage change (APC)	2016-2021 annual percentage change (APC)	P-value
African American	0.55 (0.4, 0.9)	0.59 (0.2, 1.1)	p = 0.114
Hispanic	0.3 (0.1, 0.9)	0.9 (0.4, 1.3)	p = 0.025
Whites	0.72 (0.5, 1.1)	0.77 (0.3, 1.5)	p = 0.232
Asian	0.9 (0.6, 1.5)	-1.4 (-2.1, -1.1)	p < 0.01
Other races	0.23 (0.1, 0.9)	0.25 (0.1, 1.1)	p = 0.598

## Discussion

Acute gastrointestinal bleeding is a frequent reason for hospital admission. Our research analyzes the trend in upper gastrointestinal bleeding among individuals of diverse ethnicities. Our primary objective is to examine the influence of racial and ethnic characteristics on the development of UGIB, mainly by studying common risk factors and their impact on diagnosis and treatment within specific patient populations. The study aims to encompass the various effects of racial and ethnic factors in patients presenting with UGIB to (1) comprehend the correlation between ethnicity and the cause of UGIB; (2) evaluate the role of contributing factors such as genetics, environment, culture, and healthcare accessibility; (3) determine the impact of ethnicity on UGIB diagnosis and treatment; and (4) investigate the impact of socioeconomic factors on UGIB.

Causes and risk factors

UGIB is a life-threatening condition; the risk factors include nonsteroidal anti-inflammatory drug (NSAID) use, anticoagulant use, older age, smoking, alcohol intake, and history of portal hypertension [7]. Males are more likely than females to experience UGIB, and the condition can present in various ways [8]. However, the most common causes are peptic ulcer disease (PUD), Mallory-Weiss tear, and esophageal varices [9].

PUD, which includes gastric and duodenal ulcers, is the primary cause of UGIB [9]. The bacterial infection by *Helicobacter pylori* causes most cases of PUD, leading to mucosal inflammation of the stomach and duodenum [9]. NSAID use is another significant risk factor for PUD, which can cause mucosal injury [10]. In addition, patients with PUD may experience symptoms such as dyspepsia and early satiety [9]. However, the incidence of PUD has decreased with the increased treatment of *H. pylori* infection and using proton pump inhibitors [8].

Esophageal varices are the second most common cause of UGIB and are often found in patients with decompensated liver cirrhosis [7]. Variceal hemorrhage can present as melena, hematemesis, or hematochezia [7], and Child-Pugh class B/C is a predictor of hemorrhage [11]. Gastroesophageal varices are a feared complication of decompensated liver cirrhosis, with a mortality rate of up to 30% [9]. Other potential causes of UGIB include gastrointestinal tumors and gastric cancer, which may present with symptoms such as early satiety, weight loss, melena, and hematemesis [9]. The risk factors for gastric cancer include alcohol and tobacco use [9].

Given the diverse range of potential causes for UGIB, it is essential to differentiate between symptoms and clinical presentation to identify the underlying condition and determine the appropriate treatment. Prompt and accurate diagnosis of the cause of UGIB is critical to prevent potentially life-threatening complications.

Racial and ethnic disparities in upper gastrointestinal bleeding

Differences in Risk Factors Among Racial and Ethnic Groups

Studies have found that certain racial and ethnic groups in the United States have a higher risk of developing UGIB than others. African Americans and Hispanic Americans are among those who are more likely to suffer from UGIB than Caucasians [12,13]. At the same time, Hispanics were more likely to experience bleeding from esophageal varices compared to gastroduodenal ulcers, which were more common in African Americans. Previous studies have also reported ulcers to be prominent in Asians [14,15].

Higher rates of variceal bleeding in Hispanics are attributed to their increased alcohol consumption, consistent with previous data suggesting a correlation between alcohol use and variceal bleeding [16,17]. However, Whites with a similar amount of alcohol consumption did not experience equivalent variceal bleeding as Hispanics, contrary to what the literature suggests. The incidence of UGIB can be affected by various risk factors such as underlying health conditions, genetics, environmental factors, cultural differences, and access to healthcare. Hypertension, diabetes mellitus, chronic kidney disease, and congestive heart failure are known risk factors for UGIB, as well as alcohol consumption, esophageal varices, erosive gastropathy, angiodysplasia, and chronic NSAID use [18,19].

Disparities in Diagnosis and Treatment of Upper Gastrointestinal Bleeding

Upper gastrointestinal bleeding is a severe condition that often requires hospital admission and can lead to high mortality rates [20]. Early diagnosis and timely treatment are crucial in reducing mortality rates. However, ethnic factors can affect the diagnosis and treatment plans for UGIB. Studies suggest that Black and Hispanic patients may present with more severe bleeding and require more aggressive treatment than White patients [16]. For instance, African American patients with UGIB are less likely to receive endoscopy and more likely to receive blood transfusions than non-Hispanic White patients, which may result in worse outcomes due to low socioeconomic status. They are also more likely to have longer hospital stays and higher hospital costs than other racial and ethnic groups [21].

Various factors, such as poor education status, unemployment, and limited access to healthcare facilities, make certain ethnic and racial minorities more prone to delayed diagnosis and treatment of UGIB, leading to higher morbidity and mortality rates [22]. Ethnic and racial biases can impact how healthcare providers diagnose and treat UGIB. Studies suggest that African American and Hispanic patients may be less likely to be referred for diagnostic tests or receive appropriate treatment than White patients [23].

Healthcare providers need to be aware of the potential impact of ethnic and racial factors on diagnosing and treating UGIB. Increased accessibility to healthcare facilities and fewer ethnic limitations can lead to earlier diagnosis and adequate treatment implementation. This can help ensure that all patients receive timely and appropriate care, regardless of ethnicity or race.

Impact of Socioeconomic Factors on Disparities in Upper Gastrointestinal Bleeding

The incidence and mortality rates of UGIB vary among racial and ethnic groups due to socioeconomic factors. Patients from lower socioeconomic backgrounds have increased risk factors for developing UGIB, including poor diet, increased alcohol consumption, and drug abuse [24]. Limited access to high-quality healthcare facilities and lower insurance benefits also increase risk. Education and unemployment also play a role in the incidence of UGIB [25]. Inadequate healthcare facilities delay the timely diagnosis and treatment of UGIB, leading to increased morbidity and mortality [26]. To decrease healthcare disparities, efforts need to be made to provide high-quality patient care to underserved populations, such as community-based outreach programs and the expansion of healthcare insurance policies. Increasing awareness regarding UGIB and providing high-quality care to low socioeconomic status populations is essential in reducing the incidence and mortality rates of UGIB.

Factors contributing to racial and ethnic disparities in upper gastrointestinal bleeding

UGIB is a common cause of hospital admission with high mortality rates, and the risk factors include underlying health conditions, genetics, environment, culture, and access to healthcare. For example, African Americans and Hispanics have a higher prevalence of comorbidities such as hypertension, diabetes, and chronic kidney disease, all of which increase the risk of UGIB [27-30]. Additionally, they are more likely to have *H. pylori* infection, a common cause of peptic ulcer disease and UGIB [31,32]. These factors play a significant role in the prevalence of UGIB among these ethnic groups, and more research is needed to understand the relationship between ethnicity and UGIB better.

Genetic Factors

Different racial and ethnic groups are affected by genetic factors that contribute to the development of UGIB. For example, altered drug metabolism due to cytochrome P450 enzyme system variations is more common among Asians, increasing the risk of UGIB caused by NSAIDs [33,34]. Sickle cell disease is another genetic factor that increases the risk of UGIB, affecting about one in 365 African Americans [35]. Physicians treating patients with UGIB should consider these genetic factors, as they play a significant role in the prevalence of UGIB among different racial and ethnic groups.

Environmental Factors

Environmental factors, including lifestyle choices, pollution, and occupational hazards, are critical in developing UGIB among racial and ethnic groups. Alcohol consumption is a significant factor in UGIB, particularly in Hispanics, and a diet high in spicy foods is also a contributing factor [36]. Occupational exposure to chemicals and pesticides can increase the risk of UGIB among specific populations [37]. Physicians must consider these environmental factors when treating patients at risk for UGIB.

Cultural Factors

Cultural factors contribute to the discrepancies in UGIB rates among various racial and ethnic groups. Such factors include language barriers, healthcare and illness beliefs, and traditional/alternative healing practices. For example, patients from certain groups may hold beliefs about the etiology and treatment of UGIB that diverge from Western medicine guidelines and may turn to traditional healing practices, causing delays in diagnosis and treatment or even exacerbating the condition [38]. Language barriers also affect the prevalence of UGIB, with over 20% of the US population speaking a non-English language at home, making it difficult for physicians to provide adequate care and communicate with their patients [38].

Access to Healthcare

Access to healthcare is a critical factor in increasing the risk of UGIB among certain racial and ethnic groups. For example, African Americans and Hispanics have lower health insurance rates [39]. As a result, they are less likely to receive timely medical care, leading to a higher risk of complications and mortality from UGIB. Cultural, environmental, genetic, and socioeconomic factors also contribute to disparities in UGIB. Therefore, physicians must be aware of these factors to provide appropriate care and reduce poor outcomes for these patients.

Implications of racial and ethnic disparities in upper gastrointestinal bleeding

Health Outcomes for Different Racial and Ethnic Groups

In the United States, African Americans have a higher incidence of upper GI bleeding, extended hospital stays, and higher mortality rates than Caucasians and Hispanics [4]. A Canadian study found that non-White patients, especially those of South and East Asian descent, had a higher risk of mortality from UGIB than White patients [40]. These findings highlight the need for tailored interventions to improve UGIB outcomes among diverse populations.

Economic Burden of Upper Gastrointestinal Bleeding on Different Racial and Ethnic Groups

Upper gastrointestinal bleeding is a serious and costly condition affecting individuals from different ethnic groups. In addition, studies have shown that the economic burden of UGIB varies among different ethnic groups, and it is associated with various comorbidities [20].

A study in the United States found that the average hospitalization cost for UGIB was $15,000, with higher expenses observed in patients with comorbidities such as liver disease and congestive heart failure [20]. Interestingly, the study found that African American and Hispanic patients had higher hospitalization costs than non-Hispanic White patients, despite having similar clinical characteristics [20]. This suggests that ethnic disparities in UGIB outcomes may exist and require further investigation.

Similarly, a study in the United Kingdom found that the cost of UGIB was significantly higher in South Asian patients than in White British patients [20]. The study attributed this difference to the higher incidence of *H. pylori* infection in South Asian patients, a significant risk factor for UGIB [41].

Besides the economic burden, UGIB can significantly impact a patient's quality of life and productivity. For example, a study conducted in Japan found that UGIB patients had a significantly lower health-related quality of life than the general population [42]. Additionally, patients who experienced UGIB were more likely to take time off or reduce their work hours, leading to a loss of productivity [42].

Unfortunately, some patients with UGIB, especially those from disadvantaged groups, may not receive timely interventions such as early endoscopy, which can improve outcomes. For example, one study found that Black patients with UGIB were less likely to undergo endoscopy within 24 hours of admission, the recommended standard of care [4]. Other studies have also shown that uninsured patients and those with lower socioeconomic status are less likely to receive timely endoscopy and other interventions for UGIB [4]. These findings underscore the need to improve access to timely and appropriate care for all patients with UGIB, regardless of their ethnicity or socioeconomic status.

Importance of Addressing Disparities in Upper Gastrointestinal Bleeding

Studies have highlighted disparities in the outcomes of upper gastrointestinal bleeding, with some groups experiencing higher mortality rates and adverse effects. For instance, research has shown that African American and Hispanic patients with UGIB have higher rates of in-hospital mortality than their White population with similar baseline characters. Furthermore, patients with Medicaid insurance are more likely to experience rebleeding and extended hospital stays than patients with private insurance [43].

Addressing these disparities in UGIB care and outcomes requires a multifaceted approach. Improving access to healthcare for underserved populations is a crucial first step. This can include initiatives to increase health insurance coverage, expand access to primary care, and improve transportation to medical appointments. Additionally, increasing awareness and education about UGIB among patients and healthcare providers can help ensure timely diagnosis and treatment, thereby improving outcomes.

Strategies to address racial and ethnic disparities in upper gastrointestinal bleeding

Improving Access to Healthcare

Racial and ethnic disparities in healthcare refer to differences in health outcomes and access to healthcare services based on race and ethnicity [44]. These disparities have been widely established and have negatively impacted the doctor-patient relationship, resulting in inferior medical care and outcomes [45]. Cultural competence is aimed at reducing these disparities in healthcare by identifying and acknowledging the different barriers that contribute to these disparities [46].

Addressing Cultural Barriers to Healthcare

One area where racial and ethnic disparities have been studied is gastrointestinal bleeding. For example, a study conducted during the coronavirus pandemic in 2020 found that minority populations experiencing UGIB had high mortality due to shock. There were also increased Intensive care unit (ICU) admissions, blood transfusions, and intubations associated with gastrointestinal (GI) bleeding than non-Hispanic Whites experienced, despite a lower proportion of presentation for GI bleeding [6]. Another study found that patients of lower socioeconomic status, such as people without housing, had significantly higher mortality rates and less endoscopic intervention utilization than non-homeless patients admitted for GI bleeding [47].

Addressing Socioeconomic Factors Contributing to Disparities in Upper Gastrointestinal Bleeding

Language barriers, socioeconomic restrictions, and culture-related health beliefs are common causes of racial and ethnic disparities [46]. Improving cultural competency in the healthcare system can be achieved by recruiting bilingual medical staff and making medical interpreters more accessible [46]. This targets and addresses the contrast that patients experience due to language barriers [46].

Telemedicine is a current strategy to combat disparities in the healthcare system [47]. Telemedicine involves using telecommunication to provide medical care, allowing patients with geographic and time constraints to access healthcare from the comfort of their homes [47]. Telemedicine has limitations, such as the inability to perform physical examinations and the delay in escalating care in urgent situations [47]. Despite these limitations, telemedicine provides numerous benefits that can help reduce healthcare disparities [47].

The study's retrospective design is a significant limitation, which could have introduced several biases and confounding factors. Since retrospective studies rely on data collected previously, some data may need to be incomplete or accurate, leading to potential errors in the analysis. Researchers can only control the data quality to a certain extent when relying on historical medical records. Therefore, this study's findings should be interpreted with caution, as the retrospective design can compromise the accuracy and reliability of the results.

Moreover, the study did not collect socioeconomic and literacy data, which could be essential factors that affect the study's outcome. The lack of this information limits the study's ability to identify and adjust for potential confounding variables and, therefore, may reduce the accuracy and generalizability of the study's findings.

Despite these limitations, the study provides valuable insights into trends and potential healthcare disparities in upper gastrointestinal bleeding across different races and ethnicities, highlighting the importance of identifying and addressing disparities to promote health equity. Future research can build on these findings by using a prospective design and collecting additional data to investigate further the factors influencing UGIB management and improve patient outcomes.

## Conclusions

Upper gastrointestinal bleeding is a critical medical condition that requires prompt diagnosis and treatment to prevent severe outcomes. Health equity is essential in ensuring that all patients receive equal care and access to effective treatments, regardless of race or ethnicity. Racial and ethnic disparities in UGIB management can lead to higher rates of hospitalizations, more extended hospital stays, and higher healthcare costs, significantly affecting health outcomes. Our retrospective study analyzed UGIB trends across various races and ethnicities and identified significant disparities in incidence rates between racial and ethnic groups. The study also examined the influence of racial and ethnic characteristics on the development of UGIB; evaluated the role of contributing factors such as genetics, environment, culture, and healthcare accessibility; and determined ethnicity's impact on UGIB diagnosis and treatment. Identifying risk factors more common in certain racial/ethnic groups can lead to tailored interventions for these populations, reducing disparities in UGIB management and improving overall health outcomes.
